# Using gamma-band transcranial alternating current stimulation (tACS) to improve sleep quality and cognition in patients with mild neurocognitive disorders due to Alzheimer’s disease: A study protocol for a randomized controlled trial

**DOI:** 10.1371/journal.pone.0289591

**Published:** 2023-08-04

**Authors:** Hanna Lu, Jing Li, Natalie Shu Yang, Linda Chiu Wa Lam, Suk Ling Ma, Yun Kwok Wing, Li Zhang

**Affiliations:** 1 Department of Psychiatry, The Chinese University of Hong Kong, Hong Kong SAR, China; 2 The Affiliated Brain Hospital of Guangzhou Medical University, Guangzhou, China; 3 Department of Mechanical and Automation Engineering, The Chinese University of Hong Kong, Hong Kong SAR, China; University Medical Center Goettingen, GERMANY

## Abstract

**Background:**

Sleep disturbances are highly prevalent in patients with age-related neurodegenerative diseases, which severely affect cognition and even lead to accumulated *β*-amyloid. Encouraging results from recent studies on transcranial direct current stimulation (tDCS) showed moderate positive effects on sleep quality in preclinical Alzheimer’s disease (AD). Compared to tDCS, transcranial alternating current stimulation (tACS) enables the entrainment of neuronal activity with optimized focality through injecting electric current with a specific frequency and has significant enhancement effects on slow wave activities.

**Methods and design:**

This is a randomized, double-blind, sham-controlled clinical trial comparing 40 Hz tACS with tDCS in mild neurocognitive disorders due to AD with sleep disturbances. Magnetic resonance imaging (MRI) data is used to construct personalized realistic head model. Treatment outcomes, including sleep quality, cognitive performance and saliva A*β* levels will be conducted at baseline, 4^th^ week, 8^th^ week, 12^th^ week and 24^th^ week.

**Conclusions:**

It is expected that the repeated gamma-band tACS will show significant improvements in sleep quality and cognitive functions compared to tDCS and sham tDCS. The findings will provide high-level evidence and guide further advanced studies in the field of neurodegenerative diseases and sleep medicine.

**Trial registration:**

ClinicalTrials.gov Identifier: NCT05544201.

## Introduction

Alongside the increasing life expectancy in human beings, the prevalence, severity and economic burden of age-related brain diseases are growing concerns for the families and clinicians [1]. Epidemiological studies found that about 75% Hong Kong older adults experience an overall change in sleep architecture, circadian rhythms and sleep quality [2]. Poor sleep quality can have significantly impact on cognitive functions, daily activities, quality of life, and even be implicated as a potential contributing factor in the development of age-related neurodegenerative diseases, such as Alzheimer’s disease (AD) [3, 4]. Moreover, disturbed sleep-wake cycle occurs very early in the disease course and even initiates progressive cognitive decline in the seniors at high risk of developing AD.

While sleep management in individuals with preclinical AD is certainly important, growing evidence indicates that disruption of slow-wave sleep can interfere with the strengths of specific synapses tagged as relevant memory system, adding to cognitive dysfunction in these individuals [5]. Importantly, recent research suggests that slow-wave activities (SWA) can facilitate neurons in cleaning toxic material (e.g., Amyloid-*β*, A*β*), removing it from cerebral fluids [6] and enhancing the cognition [7]. This bidirectional relationship between A*β* plaque, comprised sleep and cognitive changes highlights the possibility to reverse this process through modulating brain activities.

At present, non-pharmacological therapies for sleep disturbances with dementia are accepted as the first line of treatment in clinical guidelines [8]. However, clinical trials examining the non-pharmacological treatments are very limited and current evidence is lacking for interventions that are effective in preclinical AD patients [9]. As a form of novel technology, transcranial current stimulation (tCS) shows increasing popularity because of its diverse modalities and potential benefits on sleep quality and brain function. As a common modality of tCS, transcranial direct current stimulation (tDCS) shows positive effects on post-sleep declarative memory in AD patients [10–12]. For instance, tDCS over frontal cortex during sleep could boost the SWA in healthy old adults but showed no positive effects on memory function [13]. In contrast, a clinical trial involved 19 middle-aged patients with insomnia and found no tDCS effects on sleep architecture [14]. To date, only one study with tDCS has been conducted in preclinical AD patients [15]. They found that tDCS can increase the SWA and the sleep-mediated improvement in visuo-spatial memory. The heterogeneous results of tDCS effects on sleep quality and sleep-dependent brain function highlight that targeting specific brain oscillations (i.e., SWA) is crucial to achieve optimal benefits [16].

Different from traditional tDCS, transcranial alternating current stimulation (tACS), as an advanced modality of tCS, delivers frequency-dependent sinusoidal waveform of current that enables the entrainment of neuronal activity with optimized focality through injecting electric current with a specific frequency [17]. In brief, the proposed mechanism of tACS is hypothesized to be neural entrainment of the cortex that is reflected in neural spike alignment to the externally applied stimulation waveform [18]. Compared to tDCS, tACS with a specific frequency is more effective in triggering the endogenous slow oscillations and enhancing brain function in healthy individuals as well as in patients with brain disorders [16]. For example, tACS over left dorsolateral prefrontal cortex (DLPFC) demonstrates dose-dependent effects on slow oscillations and sleep-dependent memory consolidation [7, 19, 20]. Importantly, recent encouraging findings showed that gamma-band (i.e., 40 Hz) stimulation can ameliorate Alzheimer’s associated pathology (A*β*) and have marked effects on cognitive function in aged mice [21, 22]. The current findings of tACS effects suggest that left DLPFC, as a hub of central-executive network (CEN), not only plays a key role in modulating the sleep-related brain activities, but also involves in high-level brain function (i.e., memory and learning). The tACS effects on sleep quality could affect cognition and behavior, but the specificity and neural mechanisms of these changes are not yet well understood.

Collectively, disease-specific sleep pattern dominated by slow-wave activities (i.e., theta and delta activities) can be modulated by weak current, which sheds new light on the therapeutic advantages for sleep disturbances. Particularly, high-definition tACS (HD-tACS) utilizes four small return cathodal electrodes that proficiently limit the spread of current flow and enhance the focality with gamma-band frequency. Given the very limited therapeutic options for mild NCD patients comorbid sleep disturbances, we aim to conduct a randomized controlled trial (RCT) to examine and compare the safety, efficacy and sustainability of 40 Hz HD-tACS and HD-tDCS in the management of sleep disturbances and cognitive impairments in mild NCD-AD patients. We hypothesized that 40 Hz tACS will show significant improvements in subjective sleep quality and domain-specific cognitive functions compared to tDCS and sham tDCS.

## Material and methods

### Research design

**Fig 1:**
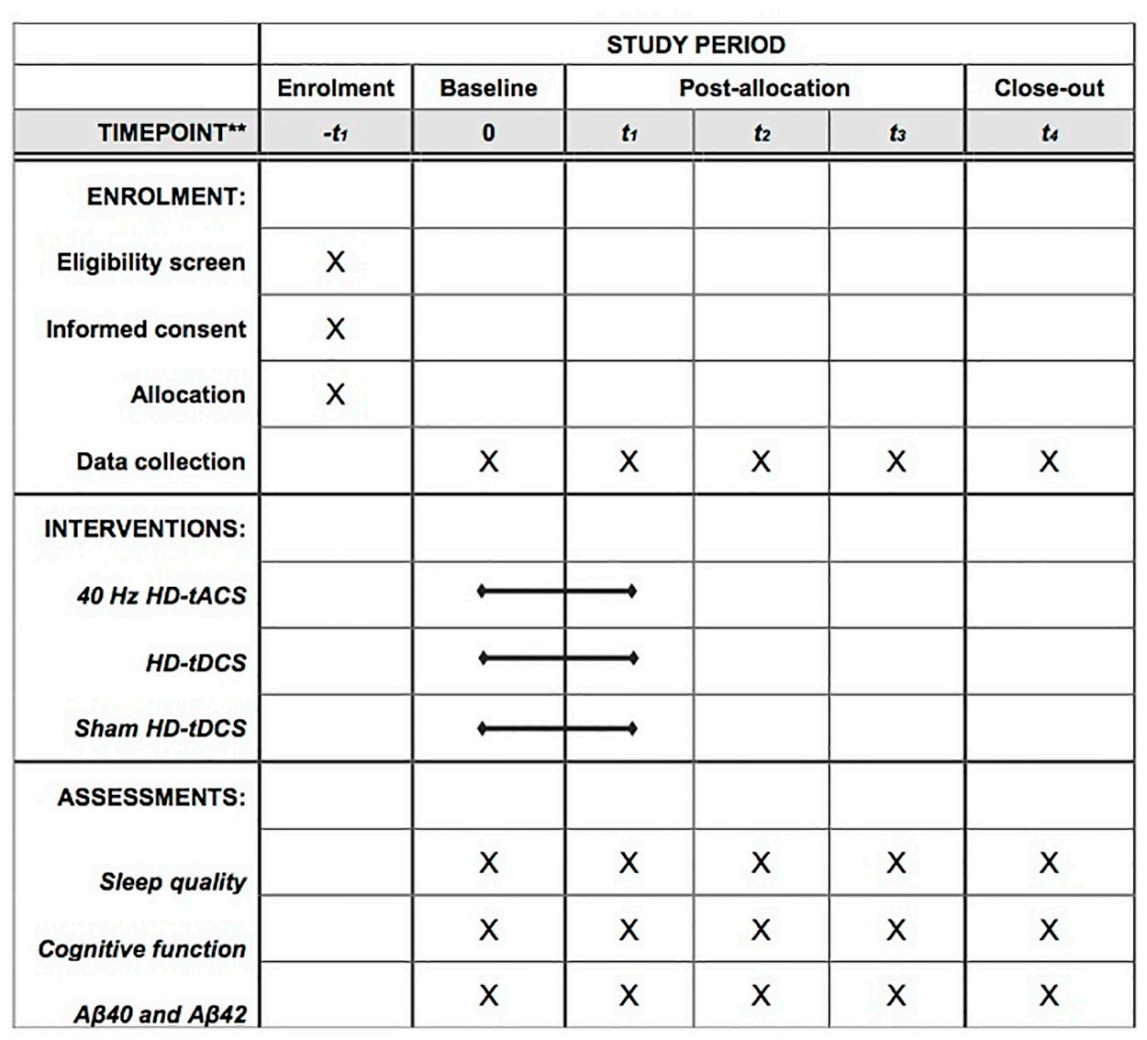
Schedule of the clinical trial according to the standard protocol items: Recommendations for interventional trials checklist (SPIRIT). Abbreviations: HD = High definition; tACS = Transcranial alternating current stimulation; tDCS = Transcranial direct current stimulation.

**Fig 2:**
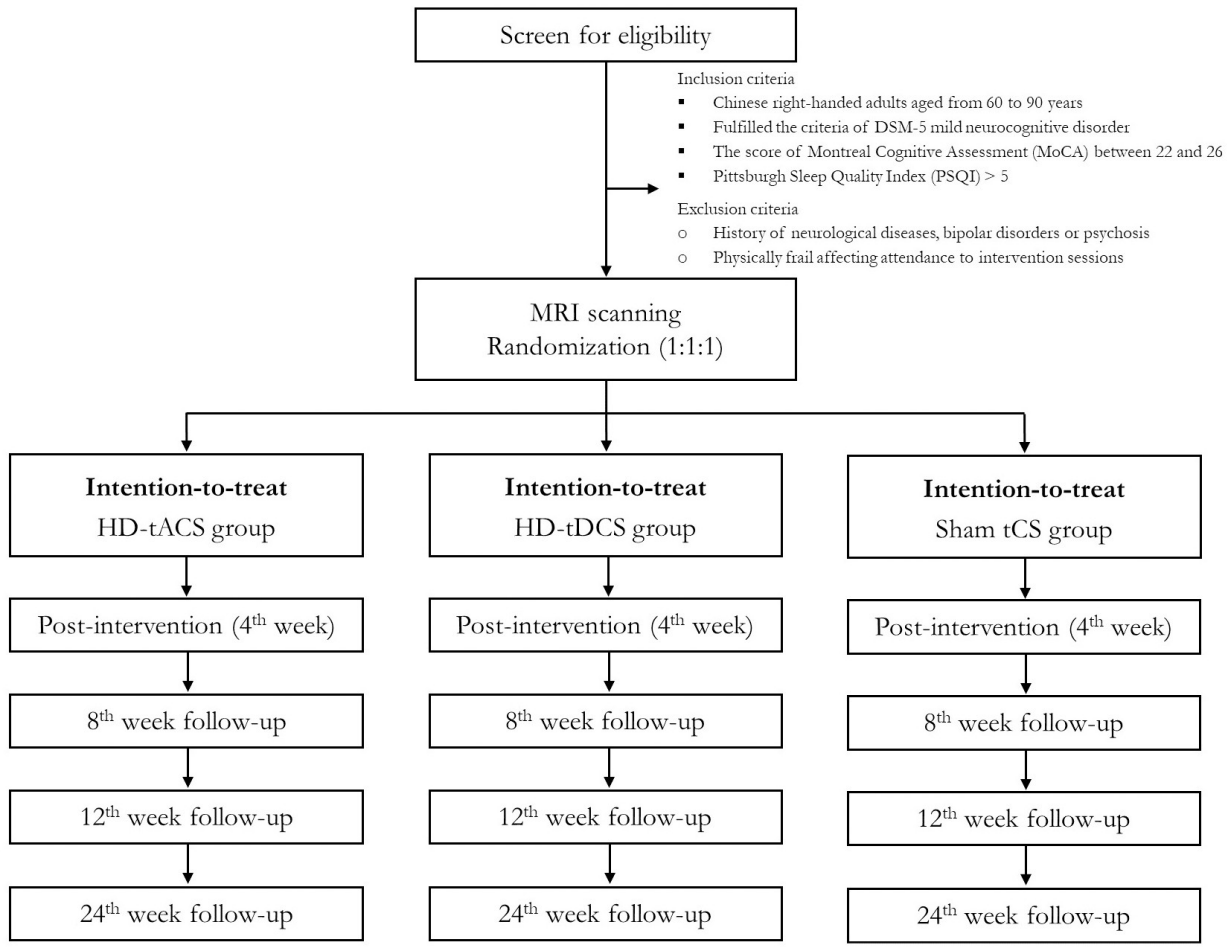
The Consolidated Standards of Reporting Trials (CONSORT) flow diagram for gamma-band transcranial alternating current stimulation (tACS) clinical trial.

This study is a randomized, double-blind (trial participants and outcomes assessors), sham-controlled clinical trial conducted at an academic cognitive training center in Hong Kong. A three-arm RCT will be carried out with repeated measures (baseline, 4^th^ week, 8^th^ week, 12^th^ week and 24^th^ week) and three conditions: 40 Hz HD-tACS, HD-tDCS and sham HD-tCS. It makes reference to the suggested requirements of a phase II design for non-pharmacological intervention. The study will be conducted following the Recommendations for Interventional Trials (SPIRIT) [23] (Fig 1) and the Consolidated Standards of Reporting Trials (CONSORT) statement (http://www.consort-statement.org) [24] (Fig 2). Written informed consent will be obtained from all participants for inclusion in the study.

### Sample size and power analysis

Until up to date no RCTs have been conducted in mild NCD-AD with sleep disturbances, therefore our calculation will be based on published data in similar population and our unpublished clinical data. Stemming from our aims, measurements would be evaluated comparing the total score of Pittsburgh Sleep Quality Index (PSQI) across time and intervention groups. The potential effect size of enhanced subjective sleep quality of tDCS is estimated from out previous findings of tDCS intervention conducted in mild neurocognitive disorder patients [25]. After 12 weeks, a 4-week course tDCS intervention demonstrated significant positive effects on the mean PSQI total score (5.364±1.5) than the other two groups (5.625±1.13; 6.2±1.3) (S1 Appendix).

Sample size is estimated using G*POWER (https://stats.idre.ucla.edu/other/gpower/). We set the confidence level as 0.95 and the desired power (1-*β*) as 0.8. Based on the proposed statistical analysis of the fixed effects of interventions on the mean PSQI total score, 28 participants in each group will be required to achieve a power of 0.8 in detecting enhancement with intervention. Accounting for an estimating of dropout rate of 15%, 33 participants for each group, a total of 99 participants, will be recruited to detect the significant treatment difference in this study.

### Study population, recruitment and eligibility criteria

Participants will be recruited through existing research cohorts, cognitive disorder clinics, and local advertisements at elderly centers in Hong Kong. The research team, including psychiatrists, sleep specialists and neuroscientists, will identify the sleep disturbances and mild neurocognitive impairments (i.e., NCD-AD). Potential participants will be invited to be screened by our trained research assistant to determine the eligibility and availability to participate in the study. Both participants and their caregivers will be briefed about the study before a decision for informed consent.

Potential participants will need to satisfy the following inclusion criteria [26]:

1. Chinese, aged from 60 to 90 years.
2. Mild neurocognitive disorder due to Alzheimer’s disease (NCD-AD) is diagnosed according to the *Diagnostic and Statistical Manual of Mental Disorders*, Fifth Edition (DSM-5) [25]. NCD-AD patients are defined by the following criteria: (1) evidence of modest cognitive decline in at least one of six domains of cognition (memory, perceptual- motor, complex attention, language, executive function and social cognition), and with clinical features indicative of AD, identified with the Montreal Cognitive Assessment (MoCA) score range from 22 to 26; (2) no interference with independence in everyday activities; (3) and no better explanation by other psychiatric disorders. NCD-AD patients fulfill the criteria of NCD and have impaired episodic memory assessed by delayed recall.
3. Sleep disturbances are defined as a total score of Pittsburgh Sleep Quality Index (PSQI) above 5 [27].

Exclusion criteria include:

1. Previous diagnosis of other major neurocognitive disorders; 2. Past history of bipolar disorders or psychosis; 3. Physically frail affecting attendance to training sessions; 4. Already attending regular training, such as cognitive behavioral therapy; 5. Taking a psychotropic or other medication known to affect cognition (e.g. anti-dementia medication); 6. Significant communicative impairments; 7. History of major neurological deficit including stroke, transient ischemic attack or brain tumor.

### Ethical issue

The study will comply with the Declaration of Helsinki and the Good Clinical Practice guidelines of the International Conference on Harmonisation of technical requirements for registration of pharmaceuticals for human use (ICH-GCP). All the participants will sign written informed consent and its conditions will be explained. Participants will be able to withdraw from the study at any time, with no consequences and without giving any reason. This study was approved by the Clinical Research Ethics Committee of The Chinese University of Hong Kong (CUHK) and New Territories East Cluster (NTEC) (The Joint CUHK-NTEC) (Date: December 8, 2020, Number: 2020.631). Besides, the study was registered in the United States National Institute of Health Registration System with Clinical Trials (Registration Number: NCT05544201).

### Pre-intervention magnetic resonance imaging

High-resolution structural MRI (sMRI) scans will be collected at the Prince of Wales Hospital using a 3.0 Tesla Philips Achieva scanner (Philips Healthcare, Best, Netherlands) within a single session during which cushioning and a thermo-plastic face mask were employed to minimize head movements. T1-weighted magnetization prepared rapid gradient echo (MPRAGE) sequence is used to optimize the grey-white contrast, with the following parameters [28]: axial acquisition with a 256×256×192 matrix, thickness = 1 mm, no gap, field of view (FOV) = 230 mm, repetition time (TR) = 2070 ms, echo time (TE) = 3.93 ms, flip angle = 15^◦^. The sequence yields high quality isotropic images with the voxel size of 1 mm ×1 mm × 1 mm. All sMRI scans will be imported to BrainSuite 14.0 (http://brainsuite.org/) for surface-based mapping. Surface-based measures include cortical thickness and folding are used as morphometric markers to precisely locate the stimulation target and predict the treatment response [29].

### Randomization, allocation and blinding

To ensure the homogeneous distribution between groups, all the eligible participants will be randomly assigned to receive: (1) tACS, (2) tDCS, (3) sham tCS following a 1:1:1 ratio. The randomization sequence is generated by a statistician who is not involved in the study design using a web-based system (http://randomization.com/). After the baseline assessment and randomization, the participants will be assigned with a trial ID number and scheduled to the corresponding intervention groups. The outcome assessors and the participants for treatment outcomes will be blinded to the grouping information. Independent research assistants who collect the inventory for sleep quality and cognitive functions will be blinded and will not participate in other outcome assessments.

### Interventions

High-definition transcranial current stimulation (HD-tCS) is delivered by a battery driven direct current stimulator (DC-Stimulator Plus, NeuroConn, Ilmenau, Germany) through a central anodal electrode surrounded by four return cathodal electrodes. The base diameter of HD-tCS electrode is 2.4 cm. We place the center electrode (i.e., anodal) over left DLPFC (i.e., F3 according to the international 10–20 EEG system) as in previous studies that investigated the transition from awake to sleep [19, 20], and place the return electrode around left DLPFC. The computational head model shows that a central anodal electrode surrounded by four return cathodal electrodes can enhance the focality of current stimulation. To ensure the electrodes are secured in place, the locations of the electrodes will be measured and positioned based on individual structural MRI. The electrodes are fixed with conductive paste (Ten20^®^, Neurodiagnostic Electrode Paste, Weaver and Company, Aurora, CO, USA). The interventions are conducted at an academic center in daytime. Participants are instructed to relax and stay wakefulness during the interventions.

### Stimulation modalities

#### High-definition transcranial alternating current stimulation (HD-tACS)

The stimulation parameters of HD-tACS include: 20 minutes at 40 Hz, 2 milliamps [20].

#### High-definition transcranial direct current stimulation (HD-tDCS)

The stimulation parameters of HD-tDCS include: 20 minutes at 2 milliamps, 20 seconds fade-in and 20 seconds fade-out [25].

#### Sham high-definition transcranial current stimulation (HD-tCS)

In sham condition, the stimulation only lasts for 30 seconds with the electrodes left in place for a further 20 minutes. This procedure mimics the transient skin sensation of tingling induced by active HD-tACS and HD-tDCS without producing any sustainable effects [25].

### Grouping and intervention schedule

All eligible participants will receive a total of 12 successive sessions of tCS intervention. According to the modalities of tCS intervention, the participants will be randomly assigned to three groups:

1. HD-tACS
2. HD-tDCS
3. Sham HD-tCS

This trial is a 4-week intervention with three sessions per week, 20 minutes per session. All participants will receive a total of 12 sessions of interventions. The schedule for intervention is the same in three randomized groups.

### Outcome measures

#### Primary outcomes

1. *Sleep quality*: The Pittsburgh Sleep Quality Index (PSQI), as a 19-item self-report questionnaire is used to evaluate the subjective sleep quality in a month [27]. The items produce seven component scores including subjective sleep quality (component 1, C1), sleep onset latency (component 2, C2), total sleep duration (component 3, C3), sleep efficiency (component 4, C4), sleep disturbances (component 5, C5), use of sleep medication (component 6, C6), and daytime dysfunction (component 7, C7). The subscore of each component ranges from 0 to 3, and the maximum total composite score of the PSQI is 21. The sum of these component scores yields a measure of global sleep quality. The cutoff score of poor sleep quality is 5 or more. This Chinese version of the PSQI has been validated with adequate reliability in cognitively intact elderly and dementia patients [30].
2. *Memory function*: Delayed recall of the words: Word-list learning test (WLLT), consisting of sixteen semantically non-associated words that is presented consecutively over three free trials of immediate recall, a 20-min delayed recall (to prevent recency effects) [31].

#### Secondary outcomes

1. Global cognition is measured by Montreal Cognitive Assessment Hong Kong version (HK MoCA), which is validated global assessment sensitive to detect early cognitive dysfunction in neurocognitive disorder [25].
2. Complex attention is measured by attention network test (ANT). The ANT paradigm (https://www.sacklerinstitute.org/cornell/assays_and_tools/ant/jin.fan/) is run by E-Prime 3.0 software [32, 33]. Within ANT paradigm, there are four types of cue: no cue, center cue, double cue, and spatial cue; and three types of flanker: neutral, congruent, and incongruent. In a given trial, a central cross-fixation point presents for 400 to 1,600 ms (randomized), subsequently is replaced for 100 ms by one of four warning cues. The target, a central arrow could appear above or below the cross-fixation and is surrounded by two flankers on each side. Before the test, all participants will be instructed to play the practice trials of ANT and required to decide whether a central arrow pointed to left or right, and press a left button if the central arrow is pointing to left, or right button if it is pointing to right. Following the illustration, participants are instructed to response as rapidly and as accurately as possible to the direction of the flanker stimulus by clicking the left or right button. After completing 24 practice trials with accurate feedback, all participants will take part in three test blocks without feedback. According to the quality control of ANT data [34], an accuracy of ANT greater than 70% indicates that the participants understand how to perform ANT and their data is qualified.
3. Executive function is measured by category verbal fluency test (CVFT). On each trial, the participants will be asked to overtly generate words in the animal category, fruit category and vegetable category as many as possible within 60 seconds. The total number of correct words is used to measure executive function [35].
4. Saliva A*β*40 and A*β*42 levels: Human saliva is an accessible and powerful diagnostic fluid for Alzheimer’s disease [36]. A*β* could be measured in saliva and can distinguish mild cognitive impairment from healthy controls [37, 38]. For each individual, saliva samples will be collected at a consistent time of day to avoid circadian effects and will be kept on ice during the collection. The levels of A*β*40 and A*β*42 in the saliva samples will be quantified by enzyme-linked immunosorbent assay (ELISA)-type assays (Aurin Biotech, Inc.). All procedures will be performed hygienically (by using disposable dental cotton rolls, microtubes, and also sterilized salivary collector tubes). Each sample will be analyzed in duplicate.
5. Quality of life and everyday functioning are measured by activities of daily living scale (ADL) [25].

### Assessment schedule

The comprehensive assessments, including sleep quality, cognitive function, saliva A*β* levels, adverse events and quality of life, will be conducted at 1 week before intervention (baseline, T0), and at 4^th^ week (T1), 8^th^ week (T2), 12^th^ week (T3) and 24^th^ week (T4).

### Statistical analysis

The data analyst will be blinded to the randomization of participants. All data will be tested for normal distribution through the Shapiro-Wilk test. Homogeneity of variance test will be used to evaluate the equality of variances among HD-tACS, HD-tDCS and sham tCS groups. Analyses will be primarily conducted by the intention-to-treat (ITT) approach. ITT means all patients who will be enrolled and randomly allocated to treatment are included in the analysis and are analyzed in the groups to which they are randomized. Linear mixed models will be used to evaluate the differences between the conditions on primary and secondary outcomes measured at each time point. This statistical method will facilitate the inclusion of the participants with missing data. Intervention, time points, and their interaction will be modelled as fixed effects. Participants will be modelled as random effects at each time point. Pre-intervention sleep quality and cognitive profiles will be compared between three randomized groups. Score changes of sleep quality, memory and global cognitive performance from baseline to follow-up points across randomized groups will be tested with occasions (time points) at level one and participants at level two. Covariates identified from baseline differences will be entered in the regression model. Secondary analyses of group differences in the performance of domain-specific cognitive functions, and its associations with between the changes of PSQI will be performed. Following statistical reporting recommendations, effect size was calculated using Cohen’s *d* for Tukey HSD post hoc tests, respectively. Statistical significance will be set at 2-sided *p* < 0.05. Computations will be performed using R Studio (version 1.1.456). We will also monitor the prevalence of adverse events and the characteristics of program adherence.

## Discussion

In this paper, we present the rationale and study protocol for a prospective, double-blind, sham-controlled randomized clinical trial. Mainly, it is expected that the individuals who receive 40 Hz HD-tACS intervention will have more improvement on subjective sleep quality and cognitive functions. It is also expected that the enhanced sleep quality and cognition will be related to decreased saliva A*β*40 and A*β*42 levels. This study stands out for a neuroscience-driven approach to address the non-pharmacological management of the common comorbidity in senior adults. Gamma-band HD-tACS has the advantage of modulating neuronal activities through frequency-specific currents, which can effectively trigger the endogenous slow oscillations (i.e., circadian rhythms) and enhance brain function, such as memory [7, 16, 19, 20].

To our best knowledge, this is the first clinical trial comparing the efficacy and sustainability of 40 Hz HD-tACS and HD-tDCS in the treatment of sleep disturbances in preclinical AD patients. In order to maintain the highest level of methodological quality, this clinical trial will be conducted following the CONSORT checklist. Participants will be randomized by a blinded assessor and will not receive the information about the differences between the interventions offered. The assessors and statistician will not have contact with the participants during the intervention period and will not have knowledge about the grouping information. In this regard, the results of this study will provide high-level clinical evidence on the utilities of modality-specific transcranial current stimulation to treat sleep disturbances in preclinical AD patients.

The results of the RCT will be circulated to international peer-reviewed journals, seminars and scientific meetings, regardless of whether the results are positive, negative or inconclusive in relation to the study hypothesis. Besides, we will also inform all participants and participants’ families about the results.

## Conclusion

The findings will provide high-level evidence and guide further advanced studies in the field of neurodegenerative diseases and sleep medicine.

## Supporting information

S1 Checklist SPIRIT 2013 checklist: Recommended items to address in a clinical trial protocol and related documents*. (PDF)

S1 File Research protocol. (ZIP)

S1 Appendix Secondary analysis of our previous transcranial direct current stimulation (tDCS) study. (PDF)
