# Supplementary material for: Using gamma-band transcranial alternating current stimulation (tACS) to improve sleep quality and cognition in patients with mild neurocognitive disorders due to Alzheimer’s disease: A study protocol for a randomized controlled trial
Source: PLoS One. 2023 Aug 4;18(8):e0289591. doi: 10.1371/journal.pone.0289591 (PMC10403094; doi:10.1371/journal.pone.0289591)
Supplement: S1 File — (ZIP) [file pone.0289591.s002.zip › Study protocol/Study Protocol.pdf]

Research Protocol

Gamma-band high-definition transcranial alternating current stimulation (HD-tACS) for sleep disturbances in mild neurocognitive disorders due to Alzheimer's disease

Department of Psychiatry, The Chinese University of Hong Kong

Department of Mechanical and Automation Engineering, The Chinese University of Hong Kong

Part I 1(c): Abstract of Research Comprehensible to a non-specialist

Background: Sleep disturbances are highly prevalent in ageing population and patients with age-related neurodegenerative diseases, which severely affect cognition and even lead to accumulated amyloid-β (Aβ). At present, non-pharmacological interventions for sleep disturbances in dementia patients are accepted as first line of treatment, of which the evidence from clinical trials is very limited. Encouraging results from recent studies on transcranial direct current stimulation (tDCS) showed moderate positive effects on sleep quality in preclinical Alzheimer's disease (AD). Compared to tDCS, high-definition transcranial alternating current stimulation (HD-tACS) enables the entrainment of neuronal activities with optimized focality through injecting small electric current with a specific frequency and has significant enhancement effects on slow wave activities.

Objectives: We aim to 1) investigate and compare the safety, efficacy and sustainability of 40 Hz HD-tACS and HD-tDCS over left dorsolateral prefrontal cortex (DLPFC) in mild neurocognitive disorder due to AD (NCD-AD) patients with sleep disturbances; 2) examine the relationship between the changes in sleep quality, cognitive function and saliva Aβ levels.

Design: A 4-week randomized, double-blind, sham-controlled trial.

Methods: Chinese right-handed mild NCD-AD patients with sleep disturbances (aged from 60 to 90 years) will be randomly assigned to a 4-week intervention of either HD-tACS, HD-tDCS, or sham HD-tCS, with 33 participants per arm. Before intervention, structural magnetic resonance imaging (MRI) data is used to construct individual realistic head model. Comprehensive assessments, including sleep quality, cognitive performance and saliva Aβ levels will be conducted at baseline, 4th week, 8th week, 12th week and 24th week. Program adherence and adverse effects will be monitored throughout intervention.

Data analysis: The primary outcomes will be the changes in sleep quality and memory performance with modality-driven paradigms (HD-tACS, HD-tDCS, sham HD-tCS), and comparisons of group differences across different time points. Secondary outcomes will be the changes objective sleep pattern, global cognition, saliva Aβ levels and quality of life. Intention-to-treat analysis will be carried out. Changes of efficacy indicators from baseline to each follow up point will be tested with mixed effect model.

Significance: This study aims to investigate the feasibility, safety and efficacy of HD-tACS and HD-tDCS over left DLPFC for sleep disturbances and cognitive dysfunction in mild NCD-AD patients. It wills also test the program adherence, tolerability and adverse effects of this innovative neurotechonology. Information will be helpful for in-depth understanding the relationship of "sleep disturbances-amyloid deposition" and guiding the further studies of sleep medicine and neurodegenerative diseases.

Part II 1 (a): Project Objectives

To investigate the effects of a 4-week 40 Hz HD-tACS on sleep quality and cognition in mild NCD-AD patients.

To examine the effects of a 4-week HD-tDCS on sleep quality and cognition in mild NCD-AD patients.

To compare the efficacy and sustainability of HD-tACS and HD-tDCS.

To examine the relationship between the changes in sleep quality, cognitive function and saliva amyloid β levels.

<u>Part II: Section 2 (a) – Background of research</u>

Alongside the increasing life expectancy in Hong Kong, the prevalence, severity and economic burden of age-related diseases are growing concerns for the families and clinicians [1]. Epidemiological studies found that about 75% Hong Kong older adults experience an overall change in sleep architecture, decrease in sleep quality, and disturbances of sleep-wake cycle [2]. More than 58% of mild dementia patients are comorbid with severe sleep problems (Lu et al., unpublished data). Poor sleep quality can have significantly impact on neurocognitive function, daily activities, quality of life, and even be implicated as a potential contributing factor in the development of age-related neurodegenerative diseases, such as Alzheimer's disease (AD) [3,4]. Moreover, disturbed sleep-wake cycle occurs very early in the disease course and even initiate progressive cognitive decline in the seniors at high risk of developing AD.

While sleep management in individuals with preclinical AD is certainly important, growing evidence indicates that disruption of slow-wave sleep can interfere with the strengths of specific synapses tagged as relevant memory system, adding to cognitive dysfunction in these individuals [5]. Importantly, recent research suggests that slow-wave activities (SWA) can facilitate neurons in cleaning toxic material (e.g., Amyloid-β, Aβ), removing it from cerebral fluids [6] and enhancing the cognition [7]. This bidirectional relationship between Aβ plaque, comprised sleep and cognitive changes highlights the possibility to reverse this process through modulating brain activities.

At present, non-pharmacological therapies for sleep disturbances with dementia are accepted as the first line of treatment in clinical guidelines [8]. However, clinical trials examining the non-pharmacological treatments are very limited and current evidence is lacking for interventions that are effective in preclinical AD [9]. As a form of novel technology, transcranial current stimulation (tCS) shows increasing popularity because of its diverse modalities and potential benefits on sleep quality and brain function.

<u>Work done by others</u>

As a common modality of tCS, transcranial direct current stimulation (tDCS) shows positive effects on post-sleep declarative memory in AD patients [10-12]. For instance, tDCS over frontal cortex during sleep could boost the SWA in healthy old adults but showed no positive effects on memory function [13]. In contrast, a clinical trial involved 19 middle-aged patients with insomnia and found no tDCS effects on sleep architecture [14]. To date, only one study with tDCS has been conducted in preclinical AD patients [15]. They found that tDCS can increase the SWA and the sleep-mediated improvement in visuo-spatial memory. The heterogenous results of tDCS effects on sleep quality and sleep-dependent brain function highlight that targeting specific brain oscillations (i.e., SWA) is crucial to achieve optimal benefits [16].

Compared to tDCS, transcranial alternating current stimulation (tACS), as an advanced modality of tCS, delivers frequency-dependent sinusoidal waveform of current that can modulate and monitor the brain activities in a more specific manner [17]. In brief, the proposed mechanism of tACS is hypothesized to be neural entrainment of the cortex that is reflected in neural spike alignment to the externally applied stimulation waveform [18]. Compared to tDCS, tACS with specific frequency is more effective in triggering the

endogenous slow oscillations and enhancing brain function in healthy individuals as well as in patients with brain disorders [16]. For example, tACS over left dorsolateral prefrontal cortex (DLPFC) shows dose-dependent effects on SWA and sleep-dependent memory consolidation [7,19,20]. Importantly, recent encouraging findings showed that gamma-band (i.e., 40 Hz) stimulation can ameliorate Alzheimer's associated pathology (Aβ) and have marked effects on cognitive function in aged mice [21,22]. The current findings of tACS effects suggest that ① left DLPFC, as a hub of central-executive network (CEN), not only plays a key role in modulating the sleep-related brain activities, but also involves in high-level brain function (i.e., executive function, memory); ② tACS effects on sleep quality can affect cognition and behavior, but the specificity (i.e., cognitive domains) and neural mechanisms of these changes are not yet well understood.

Work done by us

Our team has completed a randomized clinical trial on the cognitive effects of anodal tDCS combined with cognitive training in mild NCD-AD patients [23]. We found that a 4-week course anodal tDCS has positive effects on cognitive function. The observed tDCS-induced cognitive benefits indicate that cognitive optimization remains possible in preclinical AD. In the secondary analysis, we found that anodal tDCS has moderate positive effects on sleep quality (measured by the Pittsburgh Sleep Quality Index, PSQI).

Compared to combined modality, mild NCD-AD patients who received tDCS only demonstrated prominent enhancement on sleep quality at 12th week (Score change: F=9.5, p= 0.003, Cohen's d=0.089) (Appendix 1). Within the tDCS group (n=62), we defined poor sleepers as baseline PSQI total score>5 and good sleepers as PSQI total score≤5 [24]. After a 4-week course tDCS treatment, poor sleepers showed significantly enhanced sleep quality than good sleepers at 4th week (t=-2.41, p=0.02, Cohen's d=0.664), 8th week (t=-2.7, p=0.01, Cohen's d=0.714) and 12th week (t=-4.38, p<0.001, Cohen's d=1.209). Meanwhile, poor sleepers had more cognitive gains than good sleepers across the follow-up observations, including global cognition measured by ADAS-Cog (4th week, t=-2.42, p=0.019, Cohen's d=0.669; 8th week, t=-2.19, p=0.031, Cohen's d=0.605). Moreover, enhanced sleep quality was prominently correlated with tDCS-induced cognitive benefits (ADAS-Cog: r=0.33, p=0.029, corrected). Our results indicate that a 4-week course tDCS has significant positive effects on sleep quality and cognitive function in mild NCD-AD patients with or without sleep disturbances. Interestingly, the baseline sleep quality is a key factor that has close relationship with intervention-induced cognitive gains. Due to the lack of sham tCS (i.e., controlled group) [23], the potential placebo effects on sleep quality are not known.

Meanwhile, considering the complexity of brain waves, tACS has several advantages than tDCS: 1) Frequency-specific: tACS could generate electric current with a defined phase and frequency, which offers the opportunity to stimulate the brain region with a specific waveform; 2) Focality: the results of our computational modelling has shown that tACS could generate a more focal electric field than tDCS. 3) tACS-induced brain activity changes: stemming from our preliminary data (n=3), we found that single session of 40 Hz tACS over left DLPFC could change the brain oscillations (measured by NeuroSky head set) from alpha/beta activities (i.e., normal awake) to theta/delta activities (i.e., slow wave activities).

Collectively, disease-specific sleep pattern dominated by slow-wave activities (i.e., theta and delta activities) can be modulated by weak current, which sheds new light on the therapeutic advantages for sleep disturbances. Particularly, high-definition tACS (HD-tACS) utilizes four small return cathodal electrodes that proficiently limit the spread of current flow and enhance the focality with gamma-band frequency (Appendix 2). Given the very limited therapeutic options for mild NCD patients comorbid sleep disturbances, we aim to conduct a randomized controlled trial (RCT) to examine and compare the safety, efficacy and sustainability of 40 Hz HD-tACS and HD-tDCS in the management of sleep disturbances and cognitive impairments in mild NCD-AD patients.

Part II Section 2(b) (i) – Research plan and methodology

Research Plan

This study adopts the design of a 4-week double-blind randomized controlled trial of 40 Hz high-definition transcranial alternating current stimulation (HD-tACS). It makes reference to the suggested requirements of a phase II design for non-pharmacological intervention. The aims of this study are to explore the acceptability, potential benefits and adverse events of this novel technology. Comprehensive assessments will be conducted at the baseline, 4th week, 8th week, 12th week and 24th week to evaluate the sustainability of enhancing effects on sleep quality and cognition. Secondary outcomes include objective sleep quality, cognitive function profiles, saliva Aβ levels and quality of life.

Methodology

Recruitment centers and participants

Participants will be recruited through existing research cohorts, cognitive disorder clinics, and local advertisements at elderly centers in Hong Kong. The research team, including psychiatrists, sleep specialists and neuroscientists, will identify the sleep disturbances and mild neurocognitive impairments (i.e., NCD-AD). Potential participants will be invited to be screened by our trained research assistant to determine the eligibility and availability to participate in the study. Both participants and their caregivers will be briefed about the study before a decision for informed consent.

Potential participants will need to satisfy the following inclusion criteria [25]:

1. Chinese, aged from 60 to 90 years.

2. Mild neurocognitive disorder due to Alzheimer's disease (NCD-AD) is diagnosed according to the *Diagnostic and Statistical Manual of Mental Disorders*, Fifth Edition (DSM-5) [23]. NCD-AD patients are defined by the following criteria: (1) evidence of modest cognitive decline in at least one of six domains of cognition (memory, perceptual-motor, complex attention, language, executive function and social cognition), and with clinical features indicative of AD, identified with the Montreal Cognitive Assessment (MoCA) score range from 22 to 26; (2) no interference with independence in everyday activities; (3) and no better explanation by other psychiatric disorders. NCD-AD patients fulfill the criteria of NCD and have impaired episodic memory assessed by delayed recall.

3. Sleep disturbances are defined as a Pittsburgh Sleep Quality Index (PSQI) total score above 5 [24].

Exclusion criteria include:

1. Previous diagnosis of other major neurocognitive disorders; 2. Past history of bipolar disorders or psychosis; 3. Physically frail affecting attendance to training sessions; 4. Already attending regular training, such as cognitive behavioral therapy; 5. Taking a psychotropic or other medication known to affect cognition (e.g. anti-dementia medication); 6. Significant communicative impairments. 7. History of major neurological deficit including stroke, transient ischemic attack or brain tumor.

Pre-intervention magnetic resonance imaging

High-resolution structural MRI (sMRI) scans will be collected at the Prince of Wales Hospital using a 3.0 Tesla Philips Achieva scanner (Philips Healthcare, Best, Netherlands) within a single session during which cushioning and a thermo-plastic face mask were employed to minimize head movements. T1-weighted magnetization prepared rapid gradient echo (MPRAGE) sequence is used to optimize the grey-white contrast, with the following parameters [26]: axial acquisition with a 256×256×192 matrix, thickness = 1 mm, no gap, field of view (FOV) = 230 mm, repetition time (TR) = 2070 ms, echo time (TE) = 3.93 ms, flip angle = 15°. The sequence yields high quality isotropic images with the voxel size of 1 mm ×1 mm × 1 mm. All sMRI scans will be imported to BrainSuite 14.0 (http://brainsuite.org/) for surface-based mapping. Surface-based measures include cortical thickness and folding are used as morphometric markers to precisely locate the stimulation target and predict the treatment response [27].

Intervention schedule

This trial is a 4-week intervention with three sessions per week, 20 minutes per session. All participants will receive a total of 12 sessions of interventions. The schedule for intervention is the same in three randomized groups.

Intervention strategies

Apparatus and setting

High-definition transcranial current stimulation (HD-tCS) is delivered by a battery driven direct current stimulator (DC-Stimulator Plus, NeuroConn, Ilmenau, Germany) through a central anodal electrode surrounded by four return cathodal electrodes (Appendix 2). The base diameter of HD-tCS electrode is 2.4 cm. We place the center electrode (i.e., anodal) over left DLPFC (i.e., F3 according to the international 10-20 EEG system) as in previous studies that investigated the transition from awake to sleep [19,20], and place the return electrode around left DLPFC. The computational head model shows that a central anodal electrode surrounded by four return cathodal electrodes can enhance the focality of current stimulation (Appendix 2). To ensure the electrodes are secured in place, the locations of the electrodes will be measured and positioned based on individual structural MRI. The electrodes are fixed with conductive paste (Ten20®, Neurodiagnostic Electrode Paste, Weaver and Company, Aurora, CO, USA). Participants are instructed to relax during the setting up of transcranial current stimulation.

Stimulation modalities

High-definition transcranial alternating current stimulation (HD-tACS)

The stimulation parameters of HD-tACS include: 20 minutes at 40 Hz, 2 milliamps [20].

High-definition transcranial direct current stimulation (HD-tDCS)

The stimulation parameters of HD-tDCS include: 20 minutes at 2 milliamps, 20 seconds fade-in and 20 seconds fade-out [23].

Sham transcranial current stimulation (HD-tCS)

In sham condition, the stimulation only last for 30 seconds with the electrodes left in place for a further 20 minutes. This procedure mimics the transient skin sensation of tingling induced by active HD-tACS and HD-tDCS without producing any sustainable effects [23].

Group assignment (Appendix 3)

All eligible participants will receive a total of 12 successive sessions of tCS intervention. According to the modalities of tCS intervention, the participants will be randomly assigned to three groups:

1. HD-tACS
2. HD-tDCS
3. Sham HD-tCS

Randomization

This is a double-blind sham-controlled randomized clinical study. The assessors and the participants for clinical outcomes will be blinded to the modalities of intervention. Independent research assistants who collect the inventory for cognitive decline will be blinded and will not participate in other outcome assessments.

Outcome assessments

Primary outcomes

1. Sleep quality: The Pittsburgh Sleep Quality Index (PSQI), as a 19-item self-report questionnaire is used to evaluate the subjective sleep quality in a month [24]. The items produce seven component scores including subjective sleep quality (component 1, C1), sleep onset latency (component 2, C2), total sleep duration (component 3, C3), sleep efficiency (component 4, C4), sleep disturbances (component 5, C5), use of sleep medication (component 6, C6), and daytime dysfunction (component 7, C7). The subscore of each component ranges from 0 to 3, and the maximum total composite score of the PSQI is 21. The sum of these component scores yields a measure of global sleep quality. The cutoff score of poor sleep quality is 5 or more. This Chinese version of the PSQI has been validated with adequate reliability in cognitively intact elderly and dementia patients [29].
2. Memory function:
   Delayed recall of the words: Word-list learning test (WLLT), consisting of sixteen semantically non-associated words that is presented consecutively over three free trials of immediate recall, a 20-min delayed recall (to prevent recency effects) [23,28].

Secondary outcomes

1. Global cognition is measured by Montreal Cognitive Assessment Hong Kong version (HK MoCA), which is validated global assessment sensitive to detect early cognitive dysfunction in neurocognitive disorder [23].
2. Complex attention is measured by attention network test (ANT). The ANT paradigm (https://www.sacklerinstitute.org/cornell/assays_and_tools/ant/jin.fan/) is run by E-Prime 3.0 software [32]. Within ANT paradigm, there are four types of cue: no cue, center cue, double cue, and spatial cue; and three types of flanker: neutral, congruent, and incongruent. In a given trial, a central cross-fixation point presents for 400 to 1,600 ms (randomized), subsequently is replaced for 100 ms by one of four warning cues. The target, a central arrow could appear above or below the cross-fixation and is surrounded by two flankers on each side.
3. Executive function is measured by category verbal fluency test (CVFT). On each trial, the participants will be asked to overtly generate words in the animal category, fruit category and vegetable category as many as possible within 60 seconds. The total number of correct words is used to measure executive function [23].
4. Saliva Aβ40 and Aβ42 levels: For each individual, saliva samples will be collected at a consistent time of day to avoid circadian effects and will be kept on ice during the collection. The levels of Aβ40 and Aβ42 in the saliva samples will be quantified by enzyme-linked immunosorbent assay (ELISA)-type assays (Aurin Biotech, Inc.) [33]. Each sample will be analyzed in duplicate.
5. Quality of life and everyday functioning are measured by activities of daily living scale (ADL) [26].

<u>Assessment schedule</u>

The comprehensive assessments, including sleep quality, cognitive function, saliva Aβ levels, adverse events and quality of life, will be conducted at 1 week before intervention (baseline, T0), and at 4th week (T1), 8th week (T2), 12th week (T3) and 24th week (T4).

Sample Size Estimation

The sample size is estimated using G*POWER (https://stats.idre.ucla.edu/other/gpower/). Stemming from our aims, measurements would be evaluated comparing the PSQI total score across time and intervention groups. The potential effect size of enhanced sleep quality of tDCS is estimated from out previous findings of a tDCS intervention conducted with mild neurocognitive disorder patients (2018). After 12 weeks, a 4-week course tDCS intervention demonstrated significant positive effects on PSQI score (5.364±1.5) than the other two groups (5.625±1.13; 6.2±1.3) (Lu et al., unpublished data, Appendix 1).

We set the confidence level as 0.95 and the desired power (1-β) as 0.8. Based on the proposed statistical analysis of the fixed effects of interventions on PSQI total score, 28 participants in each group will be required to achieve a power of 0.8 in detecting enhancement with intervention. Taking into account of the dropout rate of 15%, 33 participants each group, a total of 99 participants, should be recruited in this study.

Statistical Analyses

The data analyst will be blinded to the grouping of participants. Analyses will be on an intention-to-treat basis. Linear mixed models will be used to assess the differences between conditions on the primary and secondary outcome measures at each time point. This statistical method will facilitate inclusion of participants with missing data. Intervention, time points, and their interaction will be modelled as fixed effects. Participants will be modelled as random effects at time points. Pre-intervention sleep quality and cognitive profiles will be compared between four randomized groups. Score changes of sleep quality, memory and cognitive performance from baseline to follow-up points across randomized groups will be tested with occasions (time points) at level one and participants at level two. Covariates identified from baseline differences will be entered in the regression model. Secondary analyses of group differences in outcome of domain-specific function, and association between changes of PSQI with neurocognitive function will be performed. We will also monitor the incidence of adverse events and characteristics of program adherence. Statistical significance will be set at 2-sided $p < 0.05$. Computations will be performed using R Studio (version 1.1.456).

Ethical consideration

Ethics standards will be strictly followed by providing informed consent and respecting anonymity, privacy and confidentiality. Participants will be recruited if the participants are considered mentally fit to sign consent. No personal identity, including name, birth date, mobile numbers, will be revealed in any reports or publications. Participants can withdraw anytime without interference of any future service use. For those who have any medical concerns during the study, they will be advised to seek help from clinical doctors.

Ethics approval from the Clinical Research Ethics Committee (The Joint CUHK-NTEC CREC) will be obtained before commencement of the study. The protocol will also be registered with the CCT Clinical Trials Registry of the Chinese University of Hong Kong and linked to the Clinical Trials Registry (https://clinicaltrials.gov/). The reporting of trial will follow requirements of major international journals. The study will comply with the Declaration of Helsinki and the Good Clinical Practice (GCP) guidelines of the International Conference on Harmonisation (ICH) of technical requirements for registration of pharmaceuticals for human use (ICH-GCP).

References

1. Jia, L., Quan, M., Fu, Y., Zhao, T., Li, Y., Wei, C., Shi, S. (2020). Dementia in China: epidemiology, clinical management, and research advances. The Lancet Neurology, 19(1), 81-92.

2. Chiu, H. F., Leung, T., Lam, L. C., Wing, Y. K., Chung, D. W., Li, S. W., Chi, I., Law, W. T., Boey, K. W. (1999). Sleep problems in Chinese elderly in Hong Kong. Sleep, 22(6), 717-726.

3. Ju, Y. E. S., McLeland, J. S., Toedebusch, C. D., Xiong, C., Fagan, A. M., Duntley, S. P., Morris J. C., Holtzman, D. M. (2013). Sleep quality and preclinical Alzheimer disease. JAMA neurology, 70(5), 587-593.

4. Mander, B. A., Winer, J. R., Jagust, W. J., Walker, M. P. (2016). Sleep: a novel mechanistic pathway, biomarker, and treatment target in the pathology of Alzheimer's disease?. Trends in neurosciences, 39(8), 552-566.

5. Ju, Y. E. S., Ooms, S. J., Sutphen, C., Macauley, S. L., Zangrilli, M. A., Jerome, G., Holtzman, D. M. (2017). Slow wave sleep disruption increases cerebrospinal fluid amyloid-β levels. Brain, 140(8), 2104-2111.

6. Mander, B. A., Marks, S. M., Vogel, J. W., Rao, V., Lu, B., Saletin, J. M., Fagan, A. M., Mignot, E., Zempel, J. M., Claassen, J. A. H. R., Holtzman, D. M. (2015). β-amyloid disrupts human NREM slow waves and related hippocampus-dependent memory consolidation. Nature neuroscience, 18(7), 1051.

7. Ketz, N., Jones, A., Bryant, N., Clark, V. P., Pilly, P. K. (2018). Closed-loop slow-wave tACS improves sleep dependent long-term memory generalization by modulating endogenous oscillations. Journal of Neuroscience, 0273-18.

8. Peter-Derex, L., Yammine, P., Bastuji, H., Croisile, B. (2015). Sleep and Alzheimer's disease. Sleep medicine reviews, 19, 29-38.

9. Wilfling, D., Junghans, A., Marshall, L., Eisemann, N., Meyer, G., Möhler, R., Köpke, S. (2015). Non-pharmacological interventions for sleep disturbances in people with dementia. Cochrane Database of Systematic Reviews, (9).

10. Stagg, C. J., Nitsche, M. A. (2011). Physiological basis of transcranial direct current stimulation. The Neuroscientist, 17(1), 37-53.

11. Ferrucci, R., Mameli, F., Guidi, I., Mrakic-Sposta, S., Vergari, M., Marceglia, S. E. E. A., Priori, A. (2008). Transcranial direct current stimulation improves recognition memory in Alzheimer disease. Neurology, 71(7), 493-498.

12. Bystad, M., Grønli, O., Rasmussen, I. D., Gundersen, N., Nordvang, L., Wang-Iversen, H., Aslaksen, P. M. (2016). Transcranial direct current stimulation as a memory enhancer in patients with Alzheimer's disease: a randomized, placebo-controlled trial. Alzheimer's research & therapy, 8(1), 13.

13. Paßmann, S., Külzow, N., Ladenbauer, J., Antonenko, D., Grittner, U., Tamm, S., Flöel, A. (2016). Boosting slow oscillatory activity using tDCS during early nocturnal slow wave sleep does not improve memory consolidation in healthy older adults. Brain stimulation, 9(5), 730-739.

14. Frase, L., Selhausen, P., Krone, L., Tsodor, S., Jahn, F., Feige, B., Klöppel, S. (2019). Differential effects of bifrontal tDCS on arousal and sleep duration in insomnia patients and healthy controls. Brain stimulation, 12(3), 674-683.

15. Ladenbauer, J., Ladenbauer, J., Külzow, N., de Boor, R., Avramova, E., Grittner, U., Flöel, A. (2017). Promoting sleep oscillations and their functional coupling by transcranial stimulation enhances memory consolidation in mild cognitive impairment. Journal of Neuroscience, 37(30), 7111-7124.

16. Grimaldi, D., Papalambros, N. A., Zee, P. C., Malkani, R. G. (2020). Neurostimulation techniques to enhance sleep and improve cognition in aging. Neurobiology of Disease, 104865.

17. Witkowski, M., Garcia-Cossio, E., Chander, B. S., Braun, C., Birbaumer, N., Robinson, S. E., Soekadar, S. R. (2016). Mapping entrained brain oscillations during transcranial alternating current stimulation (tACS). Neuroimage, 140, 89-98.

18. Reinhart, R. M., Nguyen, J. A. (2019). Working memory revived in older adults by synchronizing rhythmic brain circuits. Nature neuroscience, 22(5), 820-827.

19. Jones, A. P., Choe, J., Bryant, N. B., Robinson, C. S., Ketz, N. A., Skorheim, S. W., Heinrich, M. D. (2018). Dose-dependent effects of closed-loop tACS delivered during slow-wave oscillations on memory consolidation. Frontiers in Neuroscience, 12, 867.

20. Alekseichuk, I., Turi, Z., de Lara, G. A., Antal, A., Paulus, W. (2016). Spatial working memory in humans depends on theta and high gamma synchronization in the prefrontal cortex. Current Biology, 26(12), 1513-1521.

21. Iaccarino, H. F., Singer, A. C., Martorell, A. J., Rudenko, A., Gao, F., Gillingham, T. Z., Adaikkan, C. (2016). Gamma frequency entrainment attenuates amyloid load and modifies microglia. Nature, 540(7632), 230-235.

22. Martorell, A. J., Paulson, A. L., Suk, H. J., Abdurrob, F., Drummond, G. T., Guan, W., Mangena, V. (2019). Multi-sensory gamma stimulation ameliorates Alzheimer's-associated pathology and improves cognition. Cell, 177(2), 256-271.

23. Lu, H., Chan, S. S. M., Chan, W. C., Lin, C., Cheng, C. P. W., Linda Chiu Wa, L. (2019). Randomized controlled trial of TDCS on cognition in 201 seniors with mild neurocognitive disorder. Annals of clinical and translational neurology, 6, 1938-1948.

24. Chen, H. C., Hsu, N. W., Chou, P. (2020). Subgrouping poor sleep quality in community-dwelling older Adults with latent class analysis-the yilan study, taiwan. Scientific Reports, 10(1), 1-9.

25. American Psychiatric Association. (2013). Diagnostic and statistical manual of mental disorders (DSM-5®). American Psychiatric Pub.

26. Lu H, Ma SL, Chan SSM, Lam LCW (2016) The effects of apolipoprotein ε 4 on aging brain in cognitively normal Chinese elderly: a surface-based morphometry study. Int Psychogeriatr 28:1503-1511.

27. Lu, H. Quantifying age-associated cortical complexity of left dorsolateral prefrontal cortex with multiscale measurements. Journal of Alzheimer's Disease, 76, 505-516.

28. Lu H., Ni X., Fung A. W., Lam L. C. (2018). Mapping the proxies of memory and learning function in senior adults with high-performing, normal aging and neurocognitive disorders. Journal of Alzheimer's Disease, 64(3), 815-826.

29. Blackwell, T., Yaffe, K., Laffan, A., Ancoli-Israel, S., Redline, S., Ensrud, K. E., Stone, K. L. (2014). Associations of objectively and subjectively measured sleep quality with subsequent cognitive decline in older community-dwelling men: The MrOS sleep study. Sleep, 37(4), 655-663.

30. Luik, A. I., Zuurbier, L. A., Hofman, A., Van Someren, E. J., Ikram, M. A., Tiemeier, H. (2015). Associations of the 24-h activity rhythm and sleep with cognition: a population-based study of middle-aged and elderly persons. Sleep medicine, 16, 850-855.

31. Lu, H., Fung, A. W., Chan, S. S., Lam, L. C. (2016). Disturbance of attention network functions in Chinese healthy older adults: an intra-individual perspective. International psychogeriatrics, 28(2), 291-301.

32. Sabbagh, M. N., Shi, J., Lee, M., Arnold, L., Al-Hasan, Y., Heim, J., McGeer, P. (2018). Salivary beta amyloid protein levels are detectable and differentiate patients with Alzheimer's disease dementia from normal controls: preliminary findings. BMC neurology, 18(1), 1-4.
