# Supplementary material for: Using gamma-band transcranial alternating current stimulation (tACS) to improve sleep quality and cognition in patients with mild neurocognitive disorders due to Alzheimer’s disease: A study protocol for a randomized controlled trial
Source: PLoS One. 2023 Aug 4;18(8):e0289591. doi: 10.1371/journal.pone.0289591 (PMC10403094; doi:10.1371/journal.pone.0289591)
Supplement: S1 File — (ZIP) [file pone.0289591.s002.zip › Study protocol/Subject Consent Form.pdf]

Consent Form

Version 2 08-12-2021

Study Title: The effects of 40 Hz HD-tACS on sleep quality and cognition in patients with mild neurocognitive disorders due to Alzheimer's disease

Principal Investigator: Dr. Hanna Lu, The Chinese University of Hong Kong

Ethics Reference Number: 2020.631

Participant ID: <participant ID>

If you are willing to take part in this study, please read the following statements and if you agree, please sign and date overleaf.

1. I have had the opportunity to ask questions and I am satisfied with the answers and explanations provided.
2. I understand that my participant in this study is voluntary and that I am free to withdraw at any time, without giving a reason and without my medical care of legal rights being affected.
3. I understand that information from my medical records will be available to the research as part of the trial.
4. I understand that I may be randomly assigned to one treatment group, either 40 Hz HD-tACS, HD-tDCS or sham HD-tCS.
5. I understand that my unidentifiable data will be shared with other researchers.
6. I understand that I may be contacted about future, related research studies, and that I am under no obligation to take part in.
7. I agree to participate in this study.

By signing this form, you are consenting that you agree with all of the statements listed, and that the details listed below are corrects.

Name of participant： ____________

Signature of participant： _____________

Date of sign： _______________
