# Supplementary material for: Using gamma-band transcranial alternating current stimulation (tACS) to improve sleep quality and cognition in patients with mild neurocognitive disorders due to Alzheimer’s disease: A study protocol for a randomized controlled trial
Source: PLoS One. 2023 Aug 4;18(8):e0289591. doi: 10.1371/journal.pone.0289591 (PMC10403094; doi:10.1371/journal.pone.0289591)

Appendix 1. Secondary analysis of our previous transcranial direct current stimulation (tDCS) study.

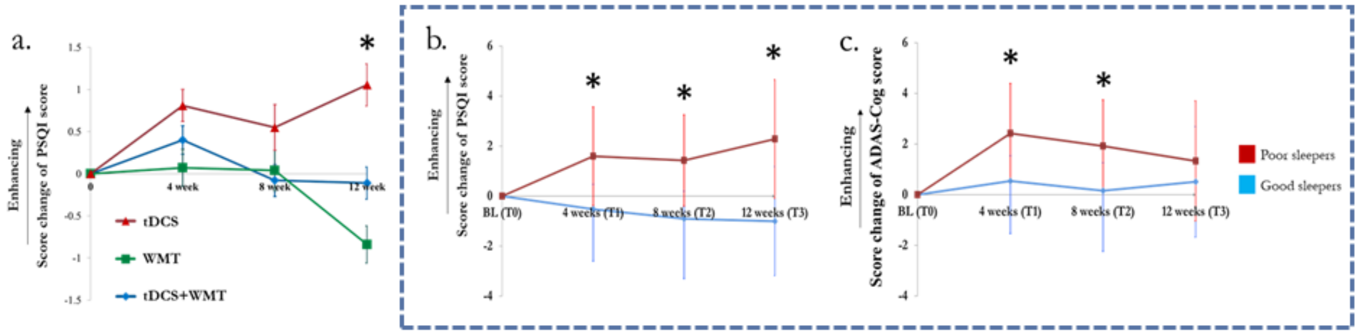

Supplement: S1 Appendix — (PDF) [file pone.0289591.s003.pdf]
